# Time-dynamic pulse modulation of spinal cord stimulation reduces mechanical hypersensitivity and spontaneous pain in rats

**DOI:** 10.1038/s41598-020-77212-w

**Published:** 2020-11-23

**Authors:** Muhammad M. Edhi, Lonne Heijmans, Kevin N. Vanent, Kiernan Bloye, Amanda Baanante, Ki-Soo Jeong, Jason Leung, Changfang Zhu, Rosana Esteller, Carl Y. Saab

**Affiliations:** 1 Department of Neurosurgery, Rhode Island Hospital, 593 Eddy St., Providence, RI 02903 USA; 2 Department of Neuroscience, Brown University, Providence, RI 02903 USA; 3 Carney Institute for Brain Science, Brown University, Providence, RI 02912 USA; 4 Boston Scientific Neuromodulation, Valencia, CA 91355 USA; 5 Department of Translational Neuroscience, School of Mental Health and Neuroscience, Maastricht University, Maastricht, The Netherlands; 6 Department of Biomedical Engineering, Cleveland Clinic, Cleveland, OH 44195 USA; 7 Department of Biomedical Engineering, Case Western Reserve University, Cleveland, OH 44106 USA

**Keywords:** Neural circuits, Neuronal physiology, Sensorimotor processing, Sensory processing, Somatosensory system

## Abstract

Enhancing the efficacy of spinal cord stimulation (SCS) is needed to alleviate the burden of chronic pain and dependence on opioids. Present SCS therapies are characterized by the delivery of constant stimulation in the form of trains of *tonic pulses* (TPs). We tested the hypothesis that modulated SCS using novel *time-dynamic pulses* (TDPs) leads to improved analgesia and compared the effects of SCS using conventional TPs and a collection of TDPs in a rat model of neuropathic pain according to a longitudinal, double-blind, and crossover design. We tested the effects of the following SCS patterns on paw withdrawal threshold and resting state EEG theta power as a biomarker of spontaneous pain: Tonic (conventional), amplitude modulation, pulse width modulation, sinusoidal rate modulation, and stochastic rate modulation. Results demonstrated that under the parameter settings tested in this study, all tested patterns except pulse width modulation, significantly reversed mechanical hypersensitivity, with stochastic rate modulation achieving the highest efficacy, followed by the sinusoidal rate modulation. The anti-nociceptive effects of sinusoidal rate modulation on EEG outlasted SCS duration on the behavioral and EEG levels. These results suggest that TDP modulation may improve clinical outcomes by reducing pain intensity and possibly improving the sensory experience.

## Introduction

Neuromodulation is defined as the alteration of nerve activity at specific neurological sites in the body through targeted delivery of a stimulus^1^. Neuromodulation therapies offer treatment options for several neurological conditions. Electrical spinal cord stimulation (SCS) is a form of neuromodulation therapy that is FDA-approved for treating mixed types of chronic and neuropathic pain conditions^2^. In the past decade, advances in SCS therapy have improved the responder rate (percent of patients that exhibit at least 50% pain reduction) from 50%^3,4^ up to 73–76% at 24 months follow up^5,6^. Despite these advances, enhancing the efficacy of SCS while improving patient experiences remains critical for ameliorating clinical outcomes and reducing the dependence on opioids for pain management.

The physiological rationale for SCS is based on the gate control theory^7^, whereby activation of large myelinated afferents inhibits nociceptive transmission in the dorsal horn. However, the mechanism of action of SCS is far more complicated and incompletely understood^8^. In conventional SCS, continuous electrical pulses are delivered with fixed parameter settings through an epidural electrode array (lead) chronically implanted over the dorsal columns and connected to a battery (pulse generator). Major parameters defining these pulses include current intensity (amplitude), frequency (rate), and pulse width. These parameters remain constant during stimulation for conventional SCS, forming* tonic* *pulse* (TP) trains. With conventional SCS, patients experience a paresthesia sensation produced by stimulation with parameter settings above perception threshold^9^. If the paresthesia intensity goes beyond tolerance, for example when postural movement or lead migration occurs^10^, patients can choose to reduce the stimulation amplitude to levels that may become sub-therapeutic. An alternative SCS modality that has provided effective pain relief is paresthesia-free stimulation where the parameters are programmed below patients’ perception level while still providing strong therapeutic benefits at amplitudes set below those evoking paresthesia, for example high-frequency stimulation (1–10 kHz)^11–13^ or bursting stimulation^14,15^. Whereas the majority of patients respond to both therapies (paresthesia and paresthesia-free), about 25% of patients may only respond to one of these modalities^16^. In addition, preference for paresthesia vs paresthesia-free therapy can vary across patients, and change over time, suggesting both modalities are important therapeutic options. Despite their therapeutic effectiveness, all of these recent advances in SCS are still deployed through *tonic* *pulse* (TP) trains, and the optimization of TPs is focused on identifying the best tonic parameter settings of the pulses for the entire stimulation.

Recently, a notable approach consisting of dynamic modulation of SCS has been explored using *time-dynamic pulses* (TDPs), whereby stimulation parameters are continuously and automatically changing based on a modulation signal^17–19^. TDPs have shown promising clinical outcomes for movement disorders such as Parkinson’s Disease^17^. Developed using a biophysical model of the dorsal horn, TDPs were described as potentially more effective than TPs for pain therapy^18^. A clinical study has shown that continuous sinusoidal modulation of pulse width provides a similar degree of pain relief compared to conventional SCS for patients with post-laminectomy syndrome^20^.

Therefore, we hypothesized that dynamic modulation of SCS parameters using TDPs at sub-threshold intensities leads to improved analgesia and sought to test and compare the SCS effects of a collection of dynamic TDP patterns in a rat model of neuropathic pain with conventional TP. The SCS patterns evaluated included five categories: (A) Tonic (conventional); (B) Amplitude Modulation; (C) Pulse Width Modulation; (D) Sinusoidal Rate Modulation; and (E) Stochastic Rate Modulation. The strengths of our approach include the use of two outcome measures of pain in rodents, the paw withdrawal threshold (mechanical hypersensitivity) and resting state EEG power in the theta (4-8 Hz) band (a biomarker of spontaneous pain^21–24^), a custom-made SCS lead for rats, and a longitudinal, randomized, double-blind, and crossover experimental design. Our data demonstrate that under the parameter settings used in this study, all the TDP patterns evaluated except pulse width modulation significantly reversed mechanical hypersensitivity. The stochastic rate modulation TDPs achieved the highest efficacy, followed by the sinusoidal rate modulation TDPs. Interestingly, the anti-nociceptive effects of sinusoidal rate modulation on EEG outlasted SCS duration. These results suggest that TDPs may improve clinical outcomes by reducing pain intensity and possibly improving the paresthesia experience.

## Results

**Figure 1:**
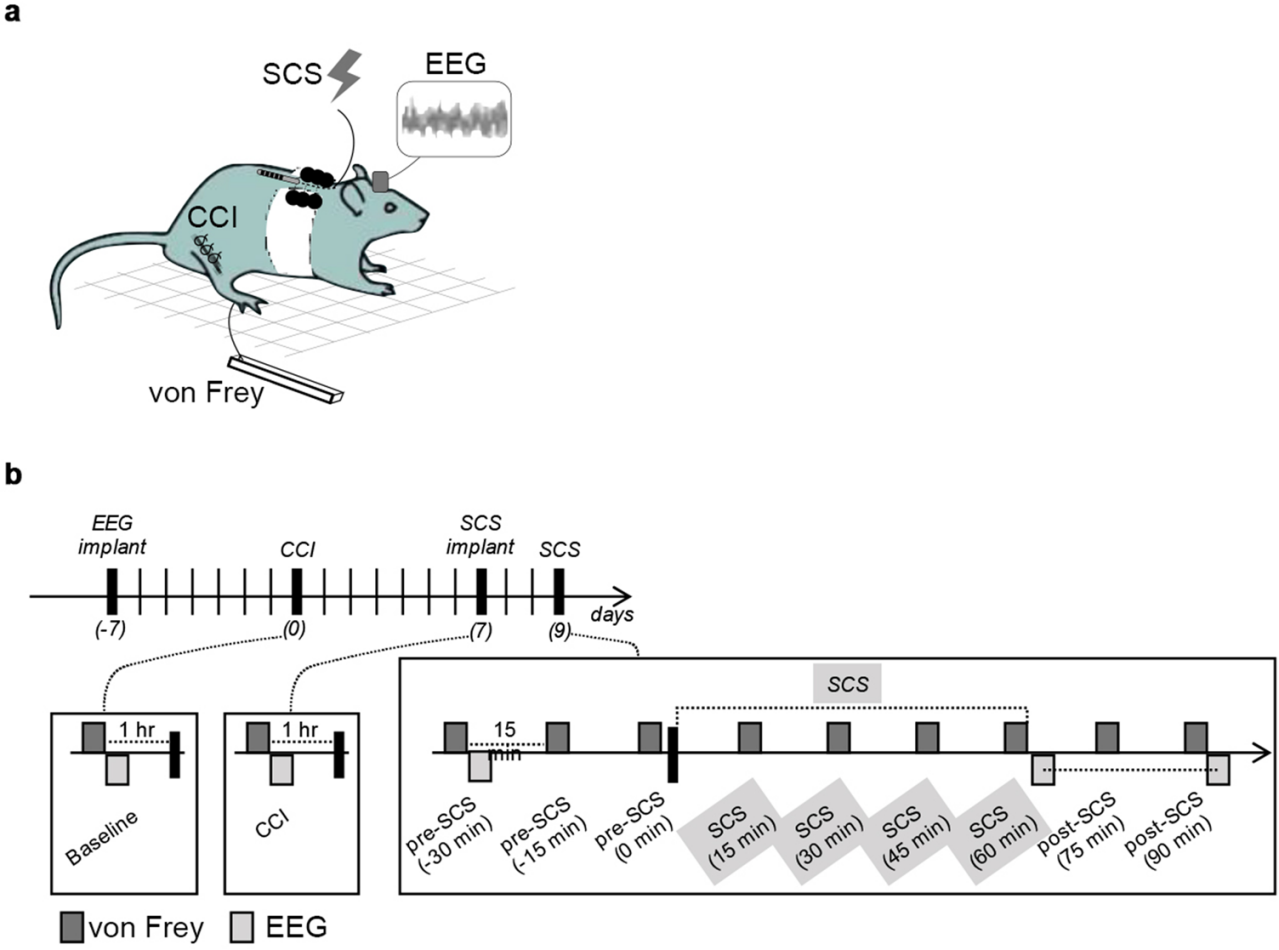
(**a**) Experimental paradigm. (**b**) Timeline of experiments. Rats first undergo implantation of EEG electrodes and are allowed one week for post-operative recovery. Second, seven days after EEG implant, CCI is done and allowed one week for post-operative recovery. Third, seven days after CCI, SCS implant is done, and allowed one day of recovery. Fourth, one day after lead implant, rats undergo SCS. For testing the analgesic effects of SCS, von Frey and EEG data are collected prior to, and at several time points during and after, SCS.

In the experiments described below, behavioral and electrophysiological data were recorded in awake rats (*n* = 8); of these, one rat was excluded from EEG analysis due to electrode failure (see Fig. 1 and “Methods” for a detailed description of the experimental set-up, procedures and timeline). Values are reported as mean ± standard error of the mean (SEM).

### CCI model validation

**Figure 2:**
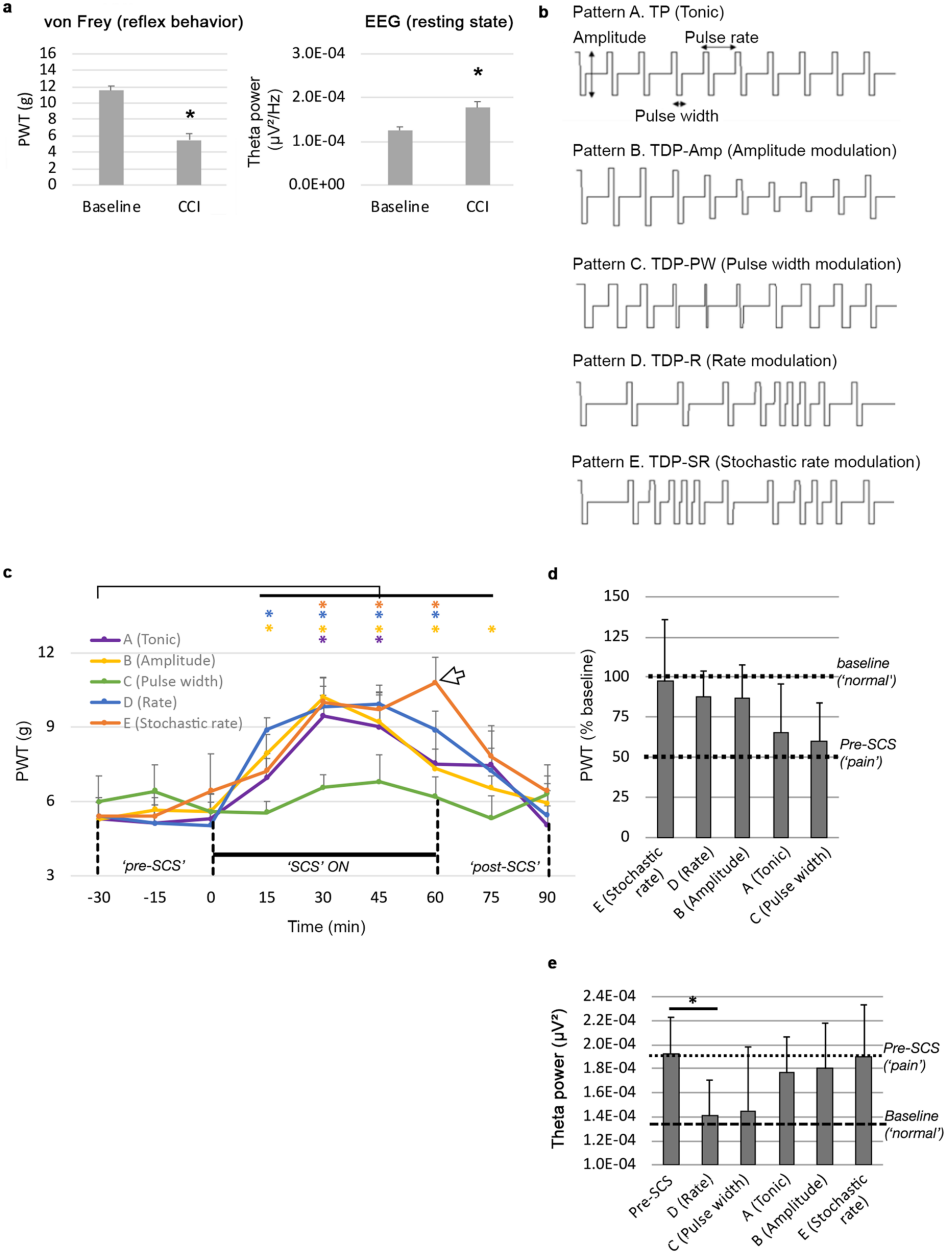
(**a**) CCI model validation. Assessment of induced chronic pain 7 days post injury using behavioral, reflex based Von Frey evaluation (n = 8) and EEG analysis (n = 5) in awake animals. Data are shown as mean ± SEM. *p < 0.05. (**b**) Schematic illustration of SCS parameters for each pattern. Pattern A: TP; Pattern B: TDP-Amp (amplitude modulation); Pattern C: TDP-PW (pulse width modulation), Pattern D: TDP-SR (rate modulation); and Pattern E: TDP-SR (stochastic rate modulation). (**c**) SCS modulation of reflex behavior. Time courses of the SCS effect on the PWT for each individual pattern (tonic, amplitude modulation, pulse width modulation, rate modulation, stochastic rate modulation) averaged for all rats. The white arrow indicates how pattern E uniquely demonstrates increases in observed PWT over time that may continue beyond the termination of SCS. (**d**) Ranking of the parameters based on their maximum SCS effects on PWT (i.e. the ratio of maximal PWT during SCS to the baseline PWT prior to CCI). Data are shown as mean ± SEM. *p < 0.05. n = 8. (**e**) SCS modulation of resting state EEG. Effects of the different SCS patterns on theta power (μV^2^) measured in awake animals during periods of rest after stimulation. Post-SCS values are averaged values of post-SCS (75) and post-SCS (90), pre-SCS values measured at − 30 min. Data are shown as mean ± SEM. *p < 0.05. n = 7.

Chronic constriction injury (CCI) is a widely used model of neuropathic pain in rats that manifests reliable symptoms of mechanical hypersensitivity measured by withdrawal behavior to von Frey monofilaments^25^. In this study (Fig. 2a, left panel), paw withdrawal threshold in the hind paw ipsilateral to CCI decreased significantly to 5.93 ± 0.76 g compared to baseline 11.58 ± 0.54 g (*p* = 0.0002, n = 8 rats), suggesting successful reproduction of the model. Previous studies from our laboratory demonstrated that spontaneous pain in rats following CCI correlates reliably with enhanced EEG theta (4–8 Hz) power in the contralateral S1^21,23,24^. In this study, results also suggest (Fig. 2a, right panel) that EEG theta power is significantly increased to 1.78E^−04^ ± 1.36E^−05^ μV^2^ compared to baseline 1.25E^−04^ ± 8.03E^−05^ μV^2^ seven days after CCI (*p* = 0.023, *n* = 5 rats). Two EEGs were excluded because of artifacts.

### SCS modulation of mechanical hypersensitivity

To assess the analgesic effects of SCS on mechanical hypersensitivity, paw withdrawal threshold (PWT) testing was performed every 15 min before, during and after SCS for each of the five SCS patterns illustrated schematically in Fig. 2b and described in Table 1. The time point when SCS was initiated was marked as t = 0 min, and the SCS was applied for a period of 60 min from t = 0 min to t = 60 min. The period between t =  − 30 min to t = 0 min was marked as pre-SCS phase while the period between t = 60 to t = 90 min was marked as post-SCS phase. The longitudinal effectiveness of each pattern was compared to the pre-SCS PWT, i.e. t =  − 30 min. Figure 2c shows a trend for the alleviation of mechanical hypersensitivity corresponding in time to SCS but to varying degrees (n = 8 rats). PWT after SCS implant was 5.48 g at t =  − 30 min compared to 5.93 g after CCI, suggesting behavioral sensitivity was unaffected by lead implant.

**Table 1:** List of SCS patterns and their constant and modulated parameters.

Setting/pattern	Pattern name	Notation	Modulated parameter	Modulation function	Constant parameter
A	Tonic	TP-Tonic	N/A	N/A	Amplitude, pulse width, rate
B	Amplitude modulation	TDP-Amp	Amplitude	Sinusoidal	Pulse width, rate
C	Pulse width modulation	TDP-PW	Pulse Width	Sinusoidal	Amplitude, rate
D	Sinusoidal rate modulation	TDP-R	Rate	Sinusoidal	Amplitude, pulse width
E	Stochastic rate modulation	TDP-SR	Rate	Stochastic	Amplitude, pulse width

Tonic (pattern A) significantly increased PWT at t = 30 min (9.46 ± 0.84 g, *p* = 0.004) and t = 45 min (9.00 ± 1.39 g, *p* = 0.049) compared to pre-SCS (5.29 ± 0.68 g). The maximal effect was achieved 30 min after initiation of SCS. SCS effectiveness is reduced at t = 60 min, whereby PWT values reverse to 7.52 ± 1.41 g, which is not significantly different from pre-SCS (p = 0.151). Similar to t = 60 min, PWT did not differ significantly from pre-SCS at t = 75 min and t = 90 min (7.45 ± 1.39 g, *p* = 0.184 and 5.04 ± 0.78 g, *p* = 0.356, respectively), suggesting transient effectiveness.

Amplitude modulation (pattern B) significantly increased PWT at t = 15 min (7.92 ± 0.79 g, *p* = 0.015), t = 30 min (10.21 ± 0.81 g, *p* = 0.002), t = 45 min (9.20 ± 1.11 g, *p* = 0.009) and t = 60 min (7.32 ± 0.80 g, *p* = 0.021) compared to pre-SCS (5.28 ± 0.69 g). The maximal effect was achieved at 30 min after initiation of SCS. SCS effectiveness is maintained at t = 75 min, whereby PWT is reduced to 6.53 ± 0.51 g but still significantly higher relative to pre-SCS (*p* = 0.034), suggesting slow therapy wash-out effects and long-term effects, but is lost at t = 90 min as PWT decreased to 5.92 ± 0.81 g (*p* = 0.348). Pulse width modulation (pattern C) showed no significant change in PWT at any time point.

Sinusoidal rate modulation (pattern D) significantly increased PWT at t = 15 min (8.90 ± 0.49 g, *p* = 0.009), t = 30 min (9.84 ± 0.82 g, *p* = 0.011), t = 45 min (9.94 ± 0.49 g, *p* = 0.006) and t = 60 min (8.89 ± 0.76 g, *p* = 0.011) compared to pre-SCS (5.41 ± 0.74 g). The maximal effect was achieved at 45 min after initiation of SCS. SCS effectiveness is reduced at t = 75 min and t = 90 min to 7.23 ± 0.93 g (*p* = 0.063) and 5.43 ± 1.01 g (*p* = 0.985), respectively.

Stochastic rate modulation (pattern E) significantly increased PWT at t = 30 min (10.02 ± 0.97 g, *p* = 0.004), t = 45 min (9.73 ± 0.97 g, *p* = 0.015) and t = 60 min (10.81 ± 1.02 g, *p* = 0.008) compared to pre-SCS (5.41 ± 0.74 g). The maximal effect was achieved at 60 min after initiation of SCS. SCS effectiveness is reduced at t = 75 min and t = 90 min to 7.83 ± 1.24 g (*p* = 0.051) and 6.43 g ± 1.05 g (*p* = 0.375), respectively.

Rankings for the maximum effects of SCS patterns on PWT are shown separately in Fig. 2d. The effect is presented as the ratio of maximal PWT during SCS to the baseline PWT prior to CCI which represents the normal status. The patterns are listed by ranking order, where the stochastic rate modulation (pattern E) ranked first with the maximal PWT reversal to 97% of the baseline, followed by the sinusoidal rate modulation (pattern D) and amplitude modulation (pattern B) which achieved PWT reversal of 88% and 87% respectively.

### SCS modulation of resting-state EEG

To assess the analgesic effects of SCS on spontaneous pain, EEG was recorded during periods of inactivity pre-SCS (t =  − 30 min) and after the SCS was terminated at t = 75 min and t = 90 min, and the power spectrum was computed (see Supplementary Fig. S1 online). Figure shows decrease in power predominantly within the theta (4–8 Hz) band compared to pre-SCS, whereas frequency in other bins is not changed. Theta power values at t = 75 min and t = 90 min were not statistically different and were therefore averaged and referred to as long-term effects (refer to timeline in Fig. 1b). Theta power post-SCS (i.e. the average of t = 75 and t = 90 min) was compared to pre-SCS.

Only stimulation with sinusoidal rate modulation (pattern D) produced significantly decreased theta power (1.41E^−04^ ± 2.98E^−05^ μV^2^) post-SCS, compared to pre-SCS (1.93E^−04^ ± 2.02E^−05^ μV^2^, *p* = 0.0189; Fig. 2e). All other stimulation patterns showed no significant change in theta power, including tonic (pattern A) (1.76E^−04^ ± 1.64E^−05^ μV^2^, *p* = 0.565), amplitude modulation (pattern B) (1.80E^−04^ ± 2.99E^−05^ μV^2^, *p* = 0.554), stochastic rate modulation (pattern E) (1.90E^−04^ ± 2.57E^−05^ μV^2^, *p* = 0.933) and pulse width modulation (pattern C) (1.45E^−04^ ± 1.45E^−05^ μV^2^, *p* = 0.060).

## Discussion

In this study, we compared the analgesic effects of conventional tonic SCS to four dynamically modulated SCS or TDPs in a rat model of neuropathic pain while providing SCS at sub-perception thresholds. Although motor thresholds are commonly used in pre-clinical models, we recently showed that motor thresholds are less reliable compared to perception thresholds because of between-subject variability^24^.We used the standard von Frey test to measure mechanical hypersensitivity and EEG to assess the effects of SCS on resting state brain oscillations and spontaneous pain^17,19–21^. While the von Frey test was administered throughout the study, EEG was only recorded when SCS was ‘off’ due to unavoidable stimulus-generated artifacts. Our results show that for the specific parameter settings evaluated in this study, all SCS patterns, except pulse width modulation, significantly reversed mechanical hypersensitivity. In the case of pulse width modulation, stimulation amplitude was set at 90% of perception threshold, while the dynamic variation of the pulse width ranged between 150 and 250 microseconds. It is possible that the perception threshold amplitude was determined at the time when the maximal pulse width was applied, therefore this amplitude could be smaller than the threshold amplitude required for smaller pulse widths. The amplitude modulation may experience similar effect from the amplitude setting, but likely to a lesser extent, because according to the strength-duration relationship^26^, the nerve fiber recruitment may be more sensitive to the pulse width variation than to the amplitude at the pulse widths used in this study. With respect to tonic SCS, the current findings replicate those of our previous study showing anti-nociceptive effects using the von Frey test in the rat CCI model^24^. However, in this study 30 min of SCS did not affect theta power, whereas in our previous study a modest reversal of theta power was observed after 4 h. of tonic SCS.

Moreover, our findings demonstrate that the sub-threshold stimulation using rate modulation TDPs led to the best results. Stochastic rate modulation TDPs achieved the highest efficacy, followed by sinusoidal rate modulation across all the TDP and TP patterns evaluated. The maximal effectiveness of the two rate modulation categories was achieved at a later time (t = 45–60 min) after initiation of SCS, as compared to that of the tonic and amplitude modulation which were achieved at t = 30 min. It should be noted that the maximal effectiveness of stochastic rate modulation was observed at t = 60 min, which is the last time point of measurement during SCS before stimulation was turned off, and the PWT was close to the “normal” baseline. It is unknown whether the PWT would continue improving and whether the improvement was going to be sustained if the SCS was applied for a longer period. The analgesic effects of sinusoidal rate modulation at the behavioral and EEG levels outlasted SCS, suggesting a longer wash-out duration and prolonged analgesic after-effects that might implicate long-term mechanisms including long-term potentiation^27^, and/or potential involvement of glial cells^28^. For patterns with fast wash-out, the EEG metric may not be as efficient to capture the pain relief produced, given that this measurement was conducted after the SCS stimulation was turned off.

It is worth noting that the parameter settings tested in this study were empirically selected without optimization, and the constant parameters and baseline setting of the modulated parameters in the TDPs (i.e. pulse width or pulse rate) were consistent with those used in the TPs. These parameter settings were strategically planned for the purpose of comparison across different TDPs and TPs without optimization of any parameter. Future studies may be carried out to further compare the effect of TPs and TDPs with parameters optimized individually for each pattern. It is also important to note that this study was implemented as a double-blind, cross-over design with the testing order randomized for all the patterns, which should have significantly reduced potential bias in the assessment, making the comparison across different patterns more robust.

Conventional epidural SCS over the dorsal columns is thought to reduce pain by ‘shutting the gate’ on nociceptive transmission^7^ while inducing paresthesia that could be preferred by some patients while unpleasant to others. The list of parameters that could be adjusted to achieve an optimal balance between analgesia and paresthesia include stimulation location, number of active electrodes, percent energy at each lead, and basic parameters of the electrical pulse (amplitude, frequency and pulse width, among others). In the last decade, the exploration in SCS for novel waveforms has primarily focused on using TPs, whose parameters are time-invariant and are typically represented by: (1) continuous electrical pulses with fixed parameters (constant amplitude, frequency, and pulse width), or (2) repeating blocks of pulses (bursts) also with fixed intra-burst and inter-burst frequency. Examples of the TP family that have also shown efficacy at intensities typically below perception threshold are the conventional tonic low-frequency SCS (~ 50 Hz) and several forms of burst^29^ and high-frequency SCS (1–10 kHz)^30–32^. An important distinction in the case of burst SCS is that the stimulation cycles between On and Off periods, whereas the intra-burst and the inter-burst parameters remain constant over time^15,29^. The majority of these aforementioned pattern categories may depend on electrical pulses that do not mimic the native neural environment, nor conform to the inherent non-stationarity of neural network dynamics where the rate of neural firing typically follows stochastic principles and varies according to temporally rich patterns^33^.

Dynamic neuromodulation has been explored in clinical settings for movement disorders with varying results^34^. One published study to date has investigated the feasibility of intensity modulation in a small sample size population of patients with post-laminectomy syndrome, resulting in a more comfortable sensation but a similar degree of pain relief compared to conventional tonic SCS^20^. Additional key observations indicate that physiologic neuronal activity in mechanical sensory systems^35,36^, or auditory system^36,37^, including the dorsal horn^35,38^, tend to be irregular and asynchronous (i.e. time-varying), with features that cannot be replicated using low pulse frequency (< 150 Hz) TPs. Although higher pulse frequency (≥ 1 kHz) TPs may produce asynchronous axon activation when applied to the dorsal column^39^, a significantly higher energy consumption is required compared to standard-of-care low frequency approaches, thus reducing device longevity and imposing an undue battery recharge burden^6^. In contrast, TDPs could better approximate stochastic sensory encoding in a more energy efficient manner compared to high frequency TPs, thereby potentially offering an approach to improving the SCS efficacy.

Amplitude and pulse width modulations are charge intensity modulation, where nerve fiber populations of different sizes may be recruited by pulses of different charge intensity in an asynchronized manner^36^, arguably mimicking the physiological mechanosensory response^40,41^. The rate-modulated pulse train may approximate the firing responses of slow adaptive afferent fibers, which conveys information about the continuity and duration of external stimulus, such as stretch or pressure. The firing rate of these afferents encodes the intensity of the stimulus^35^, therefore this type of modulation arguably induces a continuous smooth sensation with varying intensity. A major challenge in SCS therapy is the loss of effectiveness long-term for some patients^42,43^, which has been hypothesized as linked to neural adaptation to constant stimulation^44^. Therefore, therapies incorporating dynamic variations in SCS parameters may have the potential of reducing or delaying the build-up of tolerance or neural adaptation, potentially leading to therapeutic longevity.

Whereas the mechanisms of action of all SCS paradigms remain inconclusive, more recent approaches have emphasized the concepts of ‘energy delivery’ and ‘neural dosing’, whereby total charge delivery might be predictive of both clinical outcome^45^ and a dose relationship between frequency and pulse width. Frequency and charge per second has been claimed based on clinical evaluations to be a key factor to achieve optimal performance in SCS therapies applied at amplitudes below perception. The rate of charge delivery is viewed as being analogous to medication dosage, and different duty cycles might lead to distinct therapeutic benefits by allowing different energy profiles to engage neural targets in a more physiological manner, thus reducing abnormal sensation. This modern view of charge delivery, which has been validated to some extent for movement disorders, has been addressed for TPs in the neural dosing curves for pain relief that guide the selection of appropriate stimulation parameters to produce an optimal dosage for a successful SCS. Although a better understanding of dynamic modulation of SCS parameters and the independent contribution of each parameter to the therapeutic benefits of SCS is required, this study is a first step in this direction. Our findings warrant investigations into the analgesic effects of TDPs on other pain models in rodents and large animals in future studies. In summary, our pre-clinical observations suggest that dynamic modulation of selected SCS parameters using TDPs could potentially improve clinical outcomes compared to TPs.

## Methods

### Experimental animals

Adult (200–300 g) male Sprague Dawley rats were housed under a 12-h light/dark cycle in a temperature and humidity-controlled environment. Food and water were available ad libitum. All surgical procedures were performed under deep anesthesia (isoflurane, 2–2.5%). Buprenorphine Slow Release (1 mg/kg, s.c.) was administered once for post-operative pain relief secondary to EEG implant and CCI, and a half dose was administered following SCS lead implant. All the methods were carried out in accordance with the relevant guidelines and regulations, and experiments were approved by the Rhode Island Hospital Institutional Animal Care and Use Committee.

### EEG implant

As described previously^23,24,46^, the head was fixed in a stereotaxic apparatus, and the skull was exposed after a small midline skin incision. A stainless-steel screw electrode (0–80 ga 1/8-inch, impedance = 0.6 Ω; component Supply Company, Fort Meade, FL) was placed over the intact skull (i.e. epicranially), corresponding to the primary somatosensory cortex or S1 contralateral to nerve injury (Bregma − 2,2 mm lateral).

### Chronic constriction injury (CCI)

As originally described and used in our previous studies^21,24,25^, unilateral chronic constriction injury (CCI) of the sciatic nerve was used to model chronic neuropathic pain in the rat one week post-EEG implant. Four chromic gut ligatures were tied loosely around the nerve, spaced 1 mm apart. Loose ligatures were introduced as a small adjustment from typical CCI procedures to reduce the prevalence of nerve damage and deafferentation, leading to a gradual development of sensory hypersensitivity signs in rats lasting more than two weeks post-CCI. After ligation, the skin was closed with 4–0 Ethilon sutures.

### Lead implant and SCS

Seven days after CCI, a custom-designed spinal cord lead was implanted as previously described by our lab^24^ (Fig. 1a). A laminectomy was made at the T13-L1 vertebral levels and a cylindrical lead of 0.72 mm diameter with 4 contacts in linear array (Boston Scientific Neuromodulation, Valencia, CA) was inserted along the midline epidural space ending at thoracic T10–T11 levels. The lead was attached to the T13 spinous process to prevent migration and was subcutaneously tunneled, exiting the neck of the rat, where it was protected underneath a jacket. Incisions were closed using 4-0 Ethilon sutures. The rat jacket (Harvard Apparatus) is an individualized system which brings the lead to an External multi-channel stimulator system (provided by Boston Scientific Neuromodulation), as well as to an adapter circuit board. A counterweight system allowed free movement of the rat. The circuit board was connected to a commercial external stimulus generator (STG2004, Multi-Channel Systems MCS GmbH, Germany), which was used to generate the stimulation pulse trains. After one day of recovery, rats underwent stimulation sessions and behavioral testing following our randomized cross-over design (Fig. 1b). One stimulation pattern was assessed per session, which lasted 2 h and all patterns were tested in each rat. Behavioral data were collected every 15 min during the sessions and EEG data was collected after the last session preceding SCS and immediately after termination of SCS.

### SCS configuration

The stimulation configurations evaluated included five pattern categories: (A) Tonic; (B) Amplitude Modulation; (C) Pulse Width Modulation; (D) Sinusoidal Rate Modulation; and (E) Stochastic Rate Modulation (Table 1). These categories can be divided into stimulation patterns with constant pulses that do not change in time, i.e. *tonic pulse* (TP) patterns, and 4 stimulation patterns whose pulses are dynamically varying in time, i.e. *time-dynamic pulse* (TDPs) patterns. For all categories, the stimulation pulse train consisted of a sequence of pulses each defined by three main parameters: amplitude, pulse width and instantaneous rate (or period). The most typical pattern is the conventional tonic stimulation where all three pulse parameters are set to a constant value during the entire stimulation. In particular, the TP stimulation was set with a pulse width of 200 microseconds and a pulse rate of 50 Hz. Prior to assessment of each SCS category, perception threshold (PT) was estimated by ramping up the amplitude until overt behavior response to the stimulation was observed (for example attending to hindlimb or lower body, or alertness directly related to SCS onset)^24^. The stimulation amplitude was finally set to 90% of the perception threshold. For amplitude, pulse width or rate modulation TDPs, the corresponding pulse parameter was modulated with a sinusoidal function, while the other two pulse parameters were fixed. The constant parameters and baseline setting of the modulated parameters in the TDPs (refer to Table 1), were consistent with those used in the tonic stimulations, i.e. pulse width of 200 microseconds and a pulse rate of 50 Hz. The sinusoidal modulation function has a modulation frequency of 0.5 Hz, and the dynamic range of the modulated parameters were set to vary within 75–125% of the baseline for amplitude and pulse width, or within 50–150% of the baseline for the pulse rate, respectively. The stochastic rate modulation is also a modulation of instantaneous pulse rate, which followed a stochastic model instead of an explicit modulation function. The instantaneous rate of each pulse in the stochastic pulse sequence was generated using the model with an average rate of 50 Hz, which is consistent with the rate of tonic stimulation. The stimulation applied to all animals used the same collection of five pulse sequences. The TDP-SR stimulation used a pseudo-stochastic pulse sequence which was pre-generated following a stochastic model, stored, and delivered to each animal, thereby, each animal received the same sequence. We implemented a randomized cross-over design where the five stimulation patterns were applied sequentially on each rat in a randomized order. The tester and evaluator were blinded to the settings.

### EEG recording

The pin connectors of each EEG electrode were tied to a preamplifier head stage that lead to a multichannel amplifier (Iso- DAM8a; WPI Inc., Sarasota, FL). The rats were given 15 min to acclimate to the Plexiglas chamber. The EEG set-up allowed the rats to move freely within the Plexiglas chambers individually while EEG signals were recorded from S1. The EEG signals were connected to a processing system (micro 1401mkII; Cambridge Electronic Design, CED, Cambridge, United Kingdom), where they were analyzed by Spike 2 offline (CED). The EEG recordings for each session lasted 3–5 min. The sampling frequency was 16.7 kHz, down-sampled offline to 250 Hz. EEG were recorded during awake resting periods (defined as inactive wakefulness). Artifacts created from myogenic activity (for example scratching) were identified by monitoring the animal’s behavior and electrophysiology (specifically irregularities in both voltage amplitude and spectral frequency) and by applying post-hoc an automated artifact detection algorithm developed in our laboratory^47^. These artifacts were subsequently excluded from analysis.

### Paw withdrawal threshold (PWT)

Mechanical hypersensitivity was assessed by measuring the paw withdrawal thresholds (PWT) using von Frey filaments. For 5 s, each von Frey filament was applied to a hind paw according to the “up-down method”^48^. In short, filaments of increasing bending force were applied until the rat retracted its paw with a brisk withdrawal. After this positive response, a filament with a one level lower bending force was applied. A negative reaction is followed by an increase in filament bending force, while a positive reaction is followed by a decrease in filament bending force. A total of four filaments were applied after the initial positive response. The total of five responses were recorded and used to calculate the PWT.

### Statistical analysis

A repeated-measures ANOVA was used to identify statistically significant differences between observations within each parameter across various time points. One sample t-test was used to investigate statistically significant differences between Baseline and CCI values to validate the CCI model (Fig. 2a). Paired t-tests were performed between each time point compared to Pre-SCS (− 30 min) to identify statistically significant differences in PWT (Fig. 2d) and EEG (Fig. 2e). These statistical analyses utilized native functions in MATLAB and Excel. *α* < 0.05 was considered statistically significant and denoted with an asterisk (*) in relevant figures.

## Supplementary information

Supplementary Information.

Supplementary Information.

## Data Availability

The datasets generated during and/or analyzed during the current study are available from the corresponding author on reasonable request.
