# Supplementary figures and images for: Time-dynamic pulse modulation of spinal cord stimulation reduces mechanical hypersensitivity and spontaneous pain in rats
Source: Sci Rep. 2020 Nov 23;10:20358. doi: 10.1038/s41598-020-77212-w (PMC7683561; doi:10.1038/s41598-020-77212-w)

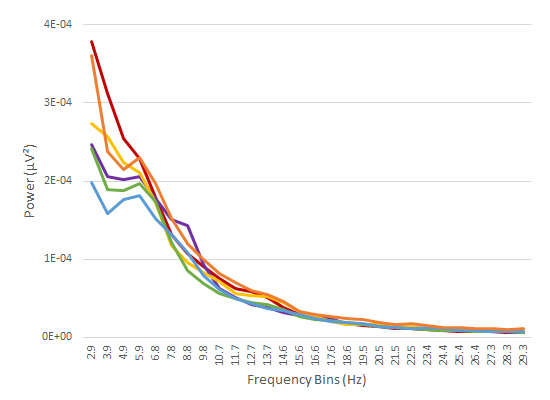

Supplement: Supplementary file 1 — Supplementary Information. [file 41598_2020_77212_MOESM1_ESM.jpg]
