# Supplementary material for: Time-dynamic pulse modulation of spinal cord stimulation reduces mechanical hypersensitivity and spontaneous pain in rats
Source: Sci Rep. 2020 Nov 23;10:20358. doi: 10.1038/s41598-020-77212-w (PMC7683561; doi:10.1038/s41598-020-77212-w)
Supplement: Supplementary file 2 — Supplementary Information. [file 41598_2020_77212_MOESM2_ESM.docx]

**Supplementary Figure 1 (S1).** Pre-SCS (n=6) and parameterized, averaged post-SCS (n=7) cortical power per frequency bin. Plots show decrease in power predominantly within the theta (4-8 Hz) band compared to pre-SCS rats, consistent with data shown in Fig 2E, whereas frequency in other bins is not changed. Post-SCS values obtained from average of available data collected at t=60 and t=90 timepoints (immediately and 30 minutes post-cessation, respectively, consistent with Figs 2C and 2E).
